# Postop QOL assessment following surgical intervention for total BPI

**DOI:** 10.1186/1753-6561-9-S3-A25

**Published:** 2015-05-19

**Authors:** Kazuteru Doi

**Affiliations:** 1 Department of Orthopaedic Surgery, Ogori Daiichi General Hospital, Ogori, Yamaguchi, 754 0002, Japan

## Introduction

Traumatic total brachial plexus injury (TTBPI) is a severe and disabling form of injury. Reconstructive modalities are complex and outcomes are variable. This is mainly because of the multitude of functions lost and relative scarcity of donor nerves to reconstruct these functions. As a result, there are several different opinions and ideologies on TTBPI.

Several papers report the functional outcomes of nerve transfers and free muscle transfers (FMT). Only few papers report retrospectively on the quality of life recovery. There are no studies which compare pre and postoperative objective and subjective parameters of quality of life.

We conducted a prospective comparative study of functional outcome and quality of life (QoL) measures of patients with TTBPI, before and after surgical treatment. The three current representative treatment modalities for elbow with/without finger function used were: double free muscle transfer (DFMT), single muscle transfer (SMT), and intercostal to musculocutaneous nerve transfers (NT).

## Method

From 2002 to 2011, Eighty-one patients, who underwent reconstructive surgery consisting of double free muscle transfer (DFMT) in 47 patients, single muscle transfer (SMT) in 16 and intercostal to muscultocutaneous nerve transfers (NT) in 17, following nerve transfer for shoulder function, were evaluated for functional outcome and QoL assessment using the Disability of Arm, Shoulder and Hand (DASH) questionnaire, pre and post-operatively for more than 24 months to be compared among three groups.

## Results

The mean shoulder abduction and flexion was comparable in all three groups, but external rotation was significantly better in the DFMT group. The range and quantitative power strength of elbow flexion were significantly larger in DFMT. Reasonable total active finger motion and power of hook grip was achieved in DFMT patients. DFMT group also showed a significant improvement in the DASH score. Amongst the 30 items of DASH, DFMT achieved superior improvement of the motor function items, but NT provided only improvement of social-mental items.

DFMT provided superior functional outcome and QoL recovery as compared to NT and SMT in patients with TTBPI.

